# Neuroimmune phenotypes in brain aneurysms and neurodegenerative diseases

**DOI:** 10.3389/fnins.2026.1916561

**Published:** 2026-09-30

**Authors:** Francis J. Lauzier, David C. Lauzier

**Affiliations:** 1 Department of Neurology, Detroit Medical Center, Wayne State University School of Medicine, Detroit, MI, United States; 2 Division of Neuropathology, University of California, Los Angeles, Los Angeles, CA, United States

**Keywords:** Alzheimer’s disease, aneurysm, immune polarization, macrophage, neurodegeneration, subarachnoid hemorrhage

## Abstract

Aneurysmal subarachnoid hemorrhage is a devastating neurological disease that occurs downstream of abnormal dilation of cerebral vessels and weakening of the vessel wall due to underlying inflammation. Vascular endothelial inflammation has been identified as a critical driver of aneurysm growth and progression, with numerous immune cell populations proposed to have a role in this process. The innate immune cells that partake in the process of aneurysm growth, primarily macrophages, can adopt pro-inflammatory or anti-inflammatory phenotypes. A recent line of work implicates an imbalance in macrophage phenotypes as a critical feature in the progression of cerebral aneurysmal disease. An imbalance in immune cell states is also observed in neurodegenerative processes, where the inflammatory phenotypes of microglia and astrocytes appear to be involved in disease progression. Understanding cerebral aneurysms and neurodegeneration as processes that involve inflammatory imbalances may provide a window for future research and identification of therapeutic targets, where modulating immune phenotypes has the potential to reduce both aneurysm rupture risk and neurodegenerative progression. Further, advances in treatments for one disease could be cautiously applied in preclinical models for the other.

## Introduction

Neurovascular inflammation is an important driver in numerous neurological diseases that elicit a high population burden, both in the United States and globally (Salvarani et al., 2007; Tian et al., 2022). Since immunotherapy has been used with success in several classes of neurological diseases, targeted investigation into neurovascular inflammation may unveil new avenues of experimental targeting that can be eventually translated into the clinical setting (Golde and Levey, 2023; Bourque and Hawiger, 2021). This may be applicable to structural cerebrovascular diseases (Lauzier et al., 2024). A cerebrovascular disease that may potentially benefit from such approaches is cerebral aneurysmal disease, which is currently treated primarily via endovascular or open surgical approaches, and currently has no disease-modifying medical therapies (Lauzier et al., 2022; Sekhar et al., 2024; Dallas et al., 2026).

Another important class of neurological diseases is neurodegenerative disease, which has numerous disease-modifying medical therapies currently used in the clinical setting, yet continues to inflict significant morbidity and mortality (Schwab et al., 2020; Valiukas et al., 2022). While many underlying drivers of neurodegenerative diseases and cerebral aneurysms are different, both involve an important inflammatory component in disease progression (Chyatte et al., 1999; Heneka et al., 2025). Indeed, imbalances in the populations of pro-inflammatory and anti-inflammatory immune effectors may underly disease progression in both classes of disease (Shao et al., 2017; Ma et al., 2024).

The various inflammatory or anti-inflammatory states of microglia and macrophages have garnered increased attention in the study of neuroimmunology (Murray and Wynn, 2011; Kapellos et al., 2019). This research has been supplemented by the identification and characterization of populations of brain-resident non-microglial macrophages known as border-associated macrophages (BAMs) (Vara-Perez and Movahedi, 2025). BAM populations include perivascular macrophages, choroid plexus macrophages, and meningeal macrophages (Silvin et al., 2023). Alongside microglia and peripheral monocyte-derived macrophages, BAMs can adopt varying inflammatory phenotypic states and complex expression profiles (Da Mesquita and Rua, 2024). The characterization of these macrophage populations has provided a new lens through which neurological disease targeting and modification can be approached. Other populations of immune cells have been observed through similar phenotypic classifications (Antuamwine et al., 2023).

In this work, we highlight the role of inflammation in both cerebral aneurysm and neurodegenerative disease, focusing our attention on immune phenotype imbalances. We concentrate our discussion of neurodegenerative diseases on Alzheimer’s disease (AD), Parkinson’s disease (PD), amyotrophic lateral sclerosis (ALS), and multiple sclerosis (MS), while cerebral aneurysms are understood to reflect the saccular morphology most frequently encountered in patients. We discuss the specific scenario of aneurysm rupture leading to subarachnoid hemorrhage (SAH), which is an upstream risk factor for the development of dementia, and provides the most conspicuous clinical link from cerebral aneurysms to neurodegeneration-like processes (Aydin and Peker, 2025). Finally, we describe the results of recent studies with pertinent therapeutic targeting.

## Immune polarization and phenotypes

After monocytes infiltrate target tissues, the local signaling milieu induces various expression profiles in monocytes that then differentiate into macrophages (Munoz-Rojas et al., 2021; Tugal et al., 2013). Though a broad range of context-specific phenotypes can be adopted, macrophages can sometimes be classified as either “pro-inflammatory” or “anti-inflammatory.” A helpful paradigm for describing these expression profiles at a higher level is the “M1/M2” schema. In this classification system, classically activated “M1” macrophages can be induced by IFN-γ, TNF-α, or LPS, whereas alternatively activated “M2” macrophages can be induced by IL-4 and IL-10 (Orecchioni et al., 2019; Mills et al., 2000). The physiological and pathological role of each “state” of macrophages is distinct. “M1” phenotypes are involved in inflammation and produce reactive oxygen species, up-regulate tissue remodeling proteases, and produce additional pro-inflammatory cytokines (Chavez-Galan et al., 2015). Conversely, “M2” phenotypes can be viewed as counteracting the M1 influence, promoting tissue healing, secreting anti-inflammatory factors, and promoting angiogenesis (Mills et al., 2000). An analogous nomenclature is sometimes used for microglia (M1/M2), astrocytes (A1/A2), and in more limited contexts, neutrophils (N1/N2), though the latter is especially controversial (Deniset and Kubes, 2018). Similarly, the utility of the A1/A2 dichotomy is more appropriately applied as a way to facilitate the understanding of reactive astrocyte states in CNS diseases (Fan and Huo, 2021).

The schema of immune polarization is broadly controversial (Ransohoff, 2016). Indeed, these represent simplifications that reflect in-vitro conditions and do not fully describe the broad transcriptomic repertoire of each cell (Paolicelli et al., 2022; Escartin et al., 2021). In reality, intermediate or even indeterminate phenotypes are present in disease states. Macrophages, the original cell population in which polarization was defined, are best described via context-dependent approaches using transcriptomic analysis techniques (Qie et al., 2022). The use of the schemas of polarity is instead useful to discuss broad, disease-level macrotrends of pro- versus anti-inflammatory cell behavior by immune effectors. Such broadening of disease descriptions allows for parallels between cerebral aneurysm and neurodegenerative disease, which otherwise have limited links, to be feasible. In the current context, the framework is more appropriately applied to microglia and macrophages, compared to other effector populations. For identification of therapeutic targets or specific mechanisms of disease, specific cellular characterization is likely warranted.

## Immune phenotypes in cerebral aneurysms

Though once considered to be congenital vessel dilations that worsen with age, saccular brain aneurysms are currently viewed as a downstream consequence of hemodynamic stress-induced inflammatory responses that cause primary vessel damage through degradation of vascular integrity (Signorelli et al., 2018). Histological studies have identified abundant inflammatory cell infiltration in cerebral aneurysms (Shimizu et al., 2019). Endoluminal vascular stress and turbulent blood flow activate the initial inflammation in the vessel wall leading to aneurysmal dilatation (Lauzier et al., 2024). This stress is responsible for the degradation of the internal elastic lamina and concurrent activation of inflammatory prostaglandin pathways in endothelial cells (Aoki et al., 2017). Local up-regulation of inflammatory cytokines results in the recruitment of circulating, blood-derived monocytes, which become macrophages once they infiltrate the vessel wall (Signorelli et al., 2018). Neutrophils may also be recruited at this stage (Ji et al., 2024). Once in the aneurysm wall, innate immune cells begin secreting IL-1β, IL-6, and TNF-α, promoting additional inflammation and subsequent vessel degradation (Shimizu et al., 2019; Aoki et al., 2009; Chalouhi et al., 2012). Of all immune cell populations, macrophages demonstrate the most pronounced infiltration in human cerebral aneurysms (Frosen et al., 2012; Chalouhi et al., 2013). Macrophages are likely important contributors to the growth and progression of brain aneurysms, as the number of macrophages present correlates with aneurysm size and growth (Shimizu et al., 2019).

A study suggest that inflammation is specifically driven by NF-kB in cerebral aneurysms, and is activated in macrophages localized in the adventitial and endothelial vessel layers, subsequently spreading throughout the entire vessel wall (Aoki et al., 2017). Pro-inflammatory functions are observed in this period of aneurysm development, with macrophages degrading the extracellular matrix through matrix metalloproteinase production, release of reactive oxygen species, and cytokine secretion into the local neurovascular milieu (Xu et al., 2019; Mitsui et al., 2020). Macrophage populations with anti-inflammatory phenotypes have also been observed in aneurysm walls (Lauzier et al., 2024). In general, “M1” and “M2” macrophages are present in similar proportions in unruptured brain aneurysms (Lauzier et al., 2024). A study found that ruptured aneurysms display elevated proportions of “M1” macrophages, therefore, periods of rapid growth leading to aneurysm rupture may be catalyzed by exaggerated inflammatory responses by macrophages themselves (Shan et al., 2021; Hasan et al., 2012). However, a more recent line of evidence suggests that an elevated M2/M1 ratio may be associated with intracranial aneurysm rupture (Stratilová et al., 2023). Another study performing single cell transcriptomic analyses in murine intracranial aneurysms identified six distinct monocyte/macrophage clusters with both pro- and anti-inflammatory features (Martinez et al., 2022). Therefore, confirmatory studies should be pursued using human tissue to identify corresponding monocyte/macrophage clusters.

## Immune phenotypes in neurodegenerative diseases

Due to their niche in the brain parenchyma, microglia have a direct influence on other glial cells and neurons, in addition to their regulatory role as the “tissue resident” macrophage population of the brain. Pro-inflammatory microglia impair oligodendrocyte function and promote inflammatory astrocyte phenotypes, in addition to being neurotoxic (Colonna and Butovsky, 2017). Conversely, anti-inflammatory microglial phenotypes secrete anti-inflammatory cytokines, perform phagocytosis, and drive tissue-restorative activities including cerebral angiogenesis (Cao and He, 2013).

The balance of inflammatory states in neurodegenerative diseases has been an area of interest in recent investigations (Guo et al., 2022). Studies suggest that AD has an early peak of anti-inflammatory microglia phenotypes, coinciding with a stage of subclinical impairment or mild cognitive impairment, followed by a pro-inflammatory peak aligning with disease progression towards more significant clinical symptoms (Fan et al., 2017; Hamelin et al., 2016). The early anti-inflammatory effects of microglia are in part mediated by the receptor TREM2, a major cell surface receptor on microglia (Rayaprolu et al., 2013). Studies have demonstrated that TREM2 overexpression improves cognitive deficits, decreases Aβ plaque deposition, and reduces M1 pro-inflammatory response through a downstream JAK/STAT/SOCS signaling pathways (Rayaprolu et al., 2013; Hansen et al., 2018). As AD progresses, a buildup of toxic amyloid products continues, which accumulates, leading to neuronal stress, inflammatory microglial polarization, and an eventual decline in clinical status (Hansen et al., 2018). Microglial polarization is also induced via ERK activation, leading to a pro-inflammatory phenotype and secretion of inflammatory cytokines, thereby causing further neuronal damage (Hansen et al., 2018; Mass et al., 2017).

A transition from anti-inflammatory to pro-inflammatory microglia (or, broadly, “M2” to “M1”) has also been reported in PD and ALS (Liao et al., 2012; Tang and Le, 2016). Initially in PD, microglia are neuroprotective, and clear accumulated α-synuclein and tau proteins, reversing oxidative stress and functional impairment (Tian et al., 2026; Scheiblich et al., 2024). As PD progresses, aggregates of α-synuclein in dead or dying dopaminergic neurons may induce a shift towards M1 microglia via NADPH oxidase activation, which increases production of reactive oxygen species and inflammatory cytokines (Zhang et al., 2005). Likewise, in ALS, experimental studies in mice have also shown a shift from M2 to M1 microglial phenotype alongside disease progression (Tang and Le, 2016). An investigation in mSOD1 mutant mice observed this shift in tandem increased expression of nitric oxide synthase and NADPH oxidase, as well as pro-inflammatory cytokines including TNF-α, IL-1β, and IL-6 (Beers et al., 2008).

MS provides an opposite broad phenotypic shift with regards to timing. Indeed, there appears to be a restorative M1 to M2 transition, where M2 microglia can release neurotrophic molecules and anti-inflammatory cytokines as remyelination occurs (Miron et al., 2013). This transition towards remyelination was noted around 10 days after the onset of the lesion, coinciding with more Arg-1 CD68^+^ M2 cells than iNOS^+^ CD68^+^ M1 cells (Miron et al., 2013). Studies suggest that this change is mediated by activin-A, a member of the TGFβ superfamily which is also involved in oligodendrocyte differentiation (Miron et al., 2013).

While some populations of microglia promote conditions favoring anti-inflammatory signaling and remyelination, prolonged M2 polarization has been linked with unwanted fibrotic response and scarring under specific CNS conditions, which could exacerbate neural injury (David and Kroner, 2011). This underscores the importance of balancing pro- and anti-inflammatory activity in neurodegenerative diseases.

## Communicating mechanisms

Aneurysmal inflammation and neurodegenerative diseases can produce a similar phenotype in the context of long-term cognitive impairment following aneurysmal SAH (Hoh et al., 2023). At the population level, a large cohort study conducted in Denmark identified an adjusted hazard ratio of 2.56 (95% CI, 2.28–2.88 in third adjusted model) for dementia after subarachnoid hemorrhage, identifying a potential relationship between the two neurological disorders (Corraini et al., 2017). There are some suggestions that delayed angiogenesis, persistent damage to capillary vessels, and chronic neuroinflammation may contribute to neurocognitive decline, though a definitive pathogenesis remains unclear (Francoeur and Mayer, 2016; Dodd et al., 2021). As the dementia following SAH is driven predominantly by vascular cognitive impairment, the overall etiology may be different, and simply produces a similar clinical phenotype. Current clinical data does not suggest that unruptured cerebral aneurysms increase the risk of developing dementia or other neurodegenerative diseases, as a 10-year observational study performed in Korea did not identify a significant difference in dementia when comparing treatment and observation for unruptured cerebral aneurysms (Han et al., 2024). This shifts the focus towards post-rupture pathological pathways. Shared vascular and systemic risk factors are known to contribute to both neurodegenerative disorders and aneurysm formation. Between the two, well-described common risk factors include hypertension, diabetes mellitus, smoking, obesity, and hypercholesterolemia (Karhunen et al., 2021; Senff et al., 2025). Hypertension, for instance, causes impaired cerebral blood flow autoregulation and microvessel disease, while advanced glycation end-products in diabetes lead to blood-brain-barrier damage (Fakih et al., 2022; Si et al., 2026; Lorenzini et al., 2024).

An additional line of evidence implicates thromboinflammation, a signaling axis linking coagulation and innate immune activation, as another pathway that may be shared between neurodegeneration and brain aneurysms (Wagner and Heger, 2022). After aneurysm rupture, thrombin generation, platelet activation, and microthrombosis amplify inflammatory signaling, in turn activating resident microglia and macrophages, and recruiting additional blood-derived leukocytes that potentially worsen brain injury (Geraghty et al., 2019). In animal models of AD and MS, the increased BBB permeability appears to allow fibrin to bind CD11b on microglia and macrophages, leading to neuroinflammatory signaling and oxidative damage that potentially exacerbate neurodegeneration (Mendiola et al., 2023; Merlini et al., 2019; Ryu et al., 2018).

## Therapeutic approaches

Specific therapeutic targeting of macrophage polarization states with a target endpoint of shifting phenotypes from “M1” to “M2” (or vice-versa) have often failed due to oversimplifications produced by the dichotomous classification system (Ransohoff, 2016). Indeed, approaching polarization as a “switch” between states does not reflect in-vivo pathological conditions. Some studies have, however, targeted specific inflammatory signaling axes in immune effectors for cerebral aneurysms and/or neurodegenerative diseases, as discussed in the following.

In brain aneurysms, shifting the macrophage polarity ratio for tissue-resident macrophages appears to impact the likelihood of aneurysm progression and rupture (Hasan et al., 2012). Limited approaches have been employed in structural cerebrovascular lesions, but candidate agents include IL-10 DNA plasmid-based nanoparticles and other aptamers to broadly shift macrophage states have been suggested, though none are used clinically (Tardito et al., 2019; Muhammad et al., 2021). Cerebral aneurysms structurally affect a small portion of the overall cerebral vasculature, and targeted delivery to the disease site provides an additional challenge. Treatments have also been explored to modulate the inflammatory responses that underly neurodegenerative disorders, and in some cases, specifically target microglia. In AD, these include inhibiting NLRP3 inflammasome-associated proteins to dampen inflammatory microglial activity, promoting 5-HT_2_A antagonism to reduce amyloid plaque deposition by promoting Aβ plaque phagocytosis by anti-inflammatory microglia, and potential enhancement of TREM2 function on microglia (Park et al., 2019; Lu et al., 2021; Schlepckow et al., 2020). Parkinson’s disease is another neurodegenerative condition in which therapeutic agents have explored modulating neuroimmune inflammatory phenotypes. Therapeutic agents including vitamin D to promote M2-like microglial phenotypes, NLRP3 inhibitors to attenuate inflammation, and GLP-1 agonists to reduce both microglial activation and alpha synuclein aggregation have been piloted (Calvello et al., 2017; Zhang et al., 2019). Additional studies have explored microglial modulation as a therapeutic target in other neurodegenerative diseases (Gao et al., 2023). For instance, myeloperoxidase inhibition in multiple system atrophy suppressed microglial activation and decreased α-synuclein aggregation, while pharmacologic CB2 receptor agonism in patients with Huntington’s disease was found to reduce neuroinflammation and striatal neuron loss (Stefanova et al., 2012; Palazuelos et al., 2009). The aforementioned therapeutic modalities are summarized in Table 1.

**Table 1:** Summary of preclinical therapeutic agents.

Class of therapeutic agent	Proposed mechanism	Disease of relevance	Animal of study	Stage of development
NLRP3 inhibitors	Inhibits exogenous seeding of tau pathology	Alzheimer’s disease	Mouse	Preclinical
5-HT2A antagonists	Reduces amyloid plaque deposition by promoting microglial Aβ phagocytosis and degradation	Alzheimer’s disease	Mouse	Preclinical
TREM2-targeting antibodies	Activates TREM2	Alzheimer’s disease	Mouse	Preclinical
Vitamin D	Inhibits microglial activation and promotes M2 polarization; protects dopaminergic neurons from inflammation and oxidative stress	Parkinson’s disease	Mouse	Preclinical and limited clinical
GLP-1 agonists	Prevents microglia-mediated astrocyte “polarization” to A1 neurotoxic phenotype	Parkinson’s disease	Mouse	Preclinical
Myeloperoxidase inhibitors	Suppresses microglial activation and neuroinflammation	Multiple System Atrophy	Mouse	Preclinical
CB2 agonists	Reduces neuroinflammation and straital neuron loss	Huntington’s disease	Mouse	Preclinical

## Conclusion and future directions

Cerebral aneurysms and neurodegenerative disorders are driven by underlying neuroinflammatory processes. Microglia and macrophages in particular are integral to these processes, with immune functional state being a key factor in disease progression. Imbalances in immune phenotypic states can contribute to neurodegenerative disorders. Therapeutics targeting these mechanisms have been explored, but have met limited success and may require additional resource investment. This study is affected by the inherent limitations of cross-disease translational discussions, and transcriptomic work assessing profiles of aneurysmal tissue and neurodegenerative processes may provide more direct evidence for identifying shared pathways and therapeutic targets.

## Data Availability

The original contributions presented in the study are included in the article/supplementary material, further inquiries can be directed to the corresponding author.
